# Zero-shot emotional speech synthesis based on feature decoupling and adaptive loss-threshold reweighting

**DOI:** 10.1371/journal.pone.0358777

**Published:** 2026-09-23

**Authors:** Yan Zhu, Yu Wang, Yijin Zhou, Bomin Liu, Rui Zhou

**Affiliations:** 1 School of Design and Art, Shanghai Dianji University, Shanghai, China; 2 School of Computer Science, Nanjing University, Nanjing, China; VIT Bhopal University, INDIA

## Abstract

Deep learning-based zero-shot speech synthesis has achieved substantial progress in speaker generalization, but stable modeling remains challenging in fine-grained emotional scenarios. Existing systems often process textual and emotional conditions through shared or closely coupled pathways, which may introduce interference between semantic content and emotional expression. In addition, uniformly averaging per-sample Conditional Flow Matching (CFM) losses may provide insufficient optimization emphasis to high-loss emotional samples. This study proposes a CFM-based zero-shot emotional speech synthesis method. Emotion–Text Decoupling Attention (ETDA) processes semantic and emotional conditions through parallel cross-attention streams and combines them through adaptive gated fusion, allowing the two conditions to retain their respective information before fusion. A 16-class fine-grained emotion space is constructed through classifier filtering and K-Means clustering based on pitch and energy features, and the resulting labels are mapped to continuous representations using a trainable lookup embedding. During training, Adaptive Loss-Threshold Reweighting estimates a threshold from mini-batch loss statistics and assigns larger weights to samples whose individual CFM losses exceed that threshold. Under speaker-disjoint evaluation on ESD, the proposed method achieves a word error rate of 3.62±0.06%, a mel-cepstral distortion of 4.45±0.05 dB, and an emotional expressiveness mean opinion score of 4.64±0.04. Controlled text-length expansion and analyses on a fixed subset of high-loss emotional samples further indicate that the proposed method maintains linguistic content and emotional expression more consistently under the evaluated conditions. These results suggest that the proposed method can, to some extent, improve content preservation, emotional expression, and acoustic reconstruction in fine-grained zero-shot emotional speech synthesis.

## 1. Introduction

Deep learning has substantially advanced acoustic modeling and high-fidelity speech generation. Prompt-conditioned zero-shot systems such as VALL-E, NaturalSpeech 2, and CosyVoice can reproduce speaker characteristics from short reference utterances and synthesize speech for speakers unseen during training [1–3]. Fine-grained emotional synthesis is more demanding because the model must preserve linguistic content, speaker identity, and intended emotion simultaneously. Its performance may also vary with utterance length, emotional complexity, and reference-prompt duration. In this study, prompt duration is controlled under matched evaluation settings, while the main focus is placed on condition interaction and sample-level optimization.

One potential limitation lies in the interaction between textual and emotional conditions. Existing systems often introduce multiple conditions through feature concatenation, shared attention, or closely coupled conditioning pathways. Although these designs facilitate information exchange, semantic and emotional cues may interact before their respective information has been sufficiently represented. This interaction may affect linguistic-content preservation and emotional consistency as the amount of generated content increases.

A second limitation concerns the optimization of emotional samples with different modeling difficulty. Standard Conditional Flow Matching (CFM) training usually averages per-sample losses within each mini-batch. This treatment assigns the same nominal coefficient to every utterance regardless of its current loss magnitude. Consequently, high-loss emotional samples may receive insufficient optimization emphasis. Existing loss-reweighting methods such as Min-SNR primarily adjust contributions across diffusion timesteps according to signal-to-noise conditions [4]. They do not directly account for differences among individual utterances within the same mini-batch.

To address the first issue, this study introduces Emotion–Text Decoupling Attention (ETDA) into the CFM acoustic decoder. ETDA computes semantic and emotional cross-attention through parallel streams and combines their outputs through adaptive gated fusion. This design retains condition-specific information before fusion while allowing frame-level adjustment of their relative contributions. To provide more specific emotional conditions, a 16-class fine-grained emotion space is constructed through classifier filtering and K-Means acoustic clustering based on pitch and energy features [5]. The resulting labels are mapped to continuous representations using a trainable lookup embedding.

To improve the optimization of high-loss emotional samples, Adaptive Loss-Threshold Reweighting is further introduced during CFM training. The strategy estimates a threshold from the mean and standard deviation of per-sample losses within each mini-batch and assigns larger weights to samples above the threshold. Samples below the threshold retain their original weights, avoiding hard exclusion from optimization. The proposed method is evaluated using an utterance-disjoint protocol on LJSpeech and a speaker-disjoint zero-shot protocol on ESD, together with objective, subjective, and mechanism-specific analyses. The main contributions of this study are summarized as follows:

1. A parallel dual-stream ETDA module is proposed to compute semantic and emotional attention independently before adaptive gated fusion, thereby reducing direct cross-modal interaction in the decoder.
2. A 16-class fine-grained emotion space is constructed through classifier filtering and K-Means acoustic clustering based on pitch and energy features. The resulting labels are mapped to continuous representations using a trainable lookup embedding.
3. Adaptive Loss-Threshold Reweighting is proposed to estimate a threshold from mini-batch loss statistics and increase the contribution of emotional samples whose individual CFM losses exceed that threshold.

The remainder of this study is organized as follows. Section 2 reviews zero-shot TTS, emotional conditioning, and loss reweighting. Section 3 presents the CFM backbone, ETDA, and Adaptive Loss-Threshold Reweighting. Section 4 describes the datasets, experimental setup, and evaluation results. Section 5 discusses the findings and limitations, and Section 6 concludes the study.

## 2. Related work

### 2.1. Zero-shot speech synthesis and cross-modal feature decoupling

Neural speech synthesis has progressively shifted from autoregressive generation toward parallel and prompt-conditioned architectures. FastPitch introduces explicit pitch prediction within a parallel Transformer, while FastSpeech 2 incorporates duration, pitch, and energy as variance information for non-autoregressive acoustic modeling [6,7]. In zero-shot synthesis, VALL-E models discrete codec tokens, CosyVoice combines supervised semantic tokens with conditional flow matching, and XTTS extends prompt-based voice cloning to multilingual settings [1,3,8]. These approaches substantially improve speaker similarity, intelligibility, and synthesis efficiency.

Their principal objective, however, is generally the reproduction of speaker identity, linguistic content, or broad speaking style from a reference prompt. When fine-grained emotional conditions are added, textual and emotional cues may still be processed through shared attention, concatenation, or closely coupled conditioning pathways, making their respective effects difficult to control independently. Existing studies have also explored speech-attribute separation. OpenVoice separates tone color from other style attributes, whereas NaturalSpeech 3 factorizes content, prosody, timbre, and acoustic details into different codec subspaces [9,10]. These studies demonstrate the value of attribute-level modeling, but they do not directly determine how textual semantics and emotional conditions should interact during decoder cross-attention. Consequently, maintaining linguistic content while preserving emotional expression remains challenging when the amount of generated content increases.

### 2.2. Controllable emotional synthesis and fine-grained emotion representation

Controllable emotional speech synthesis commonly represents emotion through categorical labels, reference utterances, natural-language descriptions, or continuous intensity variables. PromptTTS extracts style information from textual descriptions and provides an intuitive interface for controlling several speaking attributes [11]. EmoDiff uses soft-label guidance within a diffusion model to adjust categorical emotion intensity while retaining speech quality [12]. FlexiVoice further employs natural-language instructions and reference speech to control style and timbre in zero-shot synthesis [13]. These methods broaden the forms of emotional control available to users, but global prompts and utterance-level intensity values may not fully characterize local or within-class acoustic variation.

Fine-grained quantitative editing has therefore been explored at multiple temporal resolutions. Inoue et al. estimate hierarchical emotion distributions and guide emotion intensity at phoneme, word, and utterance levels [14]. Nevertheless, the reliability of fine-grained control remains closely related to the quality of the underlying emotion representation. Fixed categorical labels can obscure variation within the same nominal emotion, whereas continuous or hierarchical embeddings may depend on additional annotation, ranking models, or pretrained classifiers. Moreover, visual separation in a learned emotion space does not necessarily establish perceptual distinguishability. Constructing emotion conditions that are acoustically grounded, reproducible across speakers, and consistent with listener perception therefore remains an important issue in fine-grained emotional speech synthesis.

### 2.3. Diffusion and flow-matching models and loss reweighting

Diffusion and flow-matching models have become important approaches to acoustic generation because they provide flexible distribution modeling and high synthesis quality. Denoising Diffusion Probabilistic Models (DDPM) establish the general iterative denoising framework, and Grad-TTS adapts diffusion modeling to mel-spectrogram generation [15,16]. Flow Matching learns continuous vector fields without simulating the training trajectory, providing a basis for conditional flow-based speech generation [17]. Voicebox applies flow matching to large-scale multilingual speech generation, while Matcha-TTS develops a compact non-autoregressive architecture based on optimal-transport conditional flow matching [18,19]. NaturalSpeech 2 uses latent diffusion for zero-shot speech and singing synthesis, OZSpeech explores learned-prior-conditioned flow matching with one-step sampling, and StyleTTS 2 models expressive style using diffusion and adversarial training [2,20,21].

Although these methods differ in representation and sampling design, their training objectives are generally aggregated over samples within a mini-batch. Uniform sample averaging is straightforward, but it does not explicitly distinguish utterances according to their current reconstruction losses. Min-SNR reweights diffusion objectives according to signal-to-noise ratios across timesteps [4]; its weighting variable is therefore different from sample-level difficulty. Consequently, high-loss emotional samples may receive limited additional optimization emphasis. Sample-level reweighting based on current mini-batch statistics provides one possible way to adjust their contributions without excluding the remaining samples.

## 3. Methods

**Fig 1:**
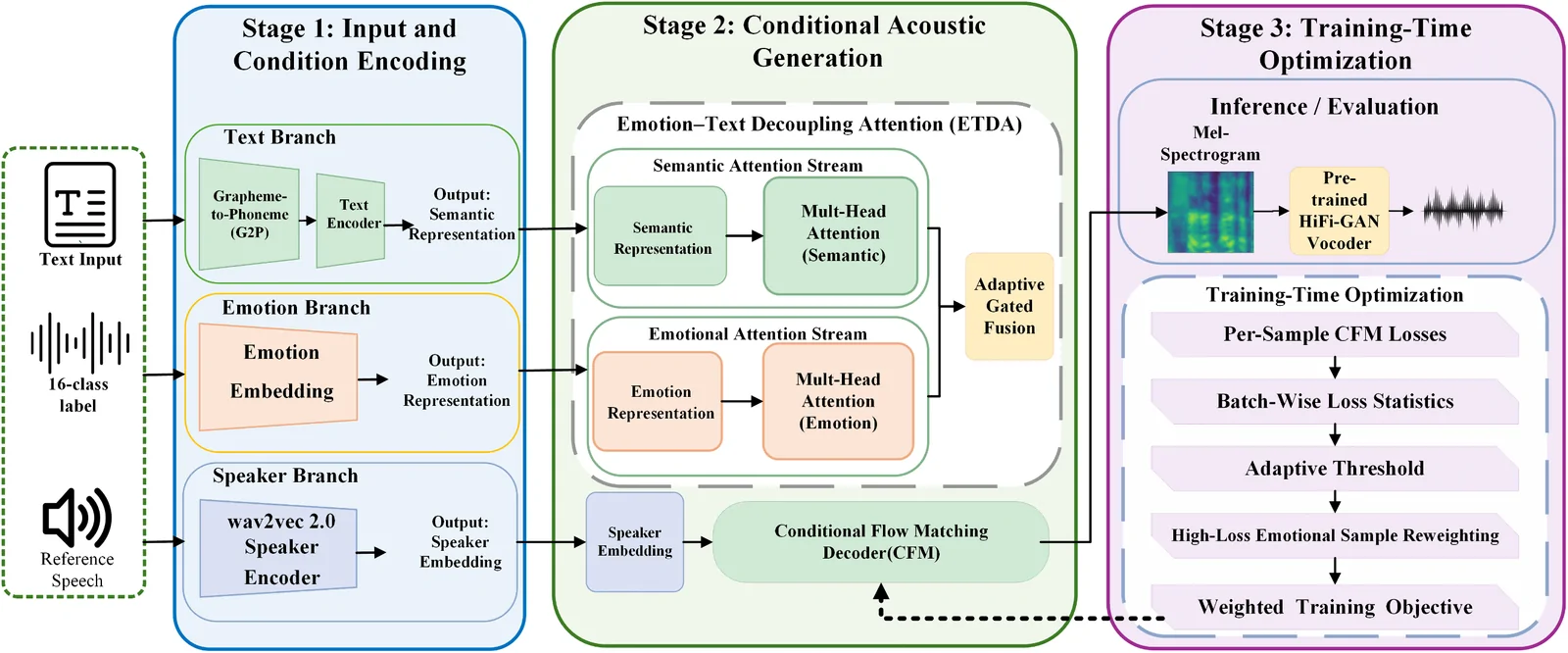
Overall architecture of the proposed method.

This study presents a Conditional Flow Matching (CFM)-based framework for fine-grained zero-shot emotional speech synthesis. As illustrated in Fig 1, the framework consists of three stages: input and condition encoding, conditional acoustic generation, and training-time optimization or inference-time waveform reconstruction. During condition encoding, the input text is converted into a semantic representation, the target emotion label is mapped to a continuous emotional representation, and the reference utterance is encoded into a speaker representation. These conditions are introduced into the acoustic decoder through different pathways.

The speaker representation provides utterance-level identity conditioning, whereas the semantic and emotional representations are processed by the proposed Emotion–Text Decoupling Attention (ETDA). ETDA employs two parallel cross-attention streams to process semantic and emotional conditions separately before adaptive gated fusion. During training, the CFM decoder is optimized using Adaptive Loss-Threshold Reweighting, which estimates a threshold from mini-batch loss statistics and increases the contribution of samples whose current losses exceed that threshold. During inference, this reweighting procedure is not used. The CFM decoder generates a mel-spectrogram, which is subsequently converted into a waveform by a pretrained HiFi-GAN vocoder.

### 3.1. Ethics statement

The listening study was approved by the Ethics Review Committee of the School of Design and Art, Shanghai Dianji University (Approval No. SDJU-SDA-ERC-2025–001; September 25, 2025). All participants provided written informed consent prior to participation. All responses were collected and analyzed anonymously.

### 3.2. Multimodal acoustic feature modeling based on conditional flow matching

The input and condition encoding stage contains three branches. In the text branch, the input text is converted into a phoneme sequence through a grapheme-to-phoneme front end and encoded as the semantic representation $c_{x}$. In the emotion branch, the fine-grained emotion label is mapped to a continuous representation $c_{e}$ using a trainable lookup embedding. In the speaker branch, a pretrained wav2vec 2.0-based encoder [22] extracts the speaker representation $c_{s}$ from the reference utterance.

The three representations are introduced into the decoder according to their functional and temporal characteristics. The utterance-level speaker representation $c_{s}$ globally conditions the acoustic features through adaptive normalization. In contrast, $c_{x}$ and $c_{e}$ are supplied to the semantic and emotional cross-attention streams of ETDA, respectively. For the *i*-th sample, the complete condition set is denoted by $c_{i}=\{c_{x,i},c_{s,i},c_{e,i}\}$.

The acoustic generation process is formulated using Conditional Flow Matching. Let $y_{i}^{*}$ denote the Ground-Truth mel-spectrogram of the *i*-th sample, and let $\epsilon_{i}$ denote Gaussian noise of the same dimensionality. For a time variable *t* sampled uniformly from [0,1], the linear conditional path and its corresponding target velocity are defined as


$$\begin{array}{c} u_{t,i}=(1-t)\epsilon_{i}+ty_{i}^{*},\;v_{t,i}^{*}=\frac{du_{t,i}}{dt}=y_{i}^{*}-\epsilon_{i}. \end{array}$$ (1)

The CFM decoder parameterized by θ estimates the conditional velocity field along this path:


$$\begin{array}{c} \frac{du_{t,i}}{dt}=v_{\theta }(u_{t,i},t|c_{i}). \end{array}$$ (2)

The per-sample CFM objective minimizes the mean squared error between the predicted and target velocities:


$$\begin{array}{c} L_{i}^{CFM}={𝔼}_{t,\epsilon_{i}}\left[{\left\|v_{\theta }(u_{t,i},t|c_{i})-v_{t,i}^{*}\right\|}_{2}^{2}\right]. \end{array}$$ (3)

Eq. (3) retains the loss at the sample level rather than directly averaging it across the mini-batch. During inference, the learned probability-flow ordinary differential equation is numerically integrated from the initial noise distribution toward the acoustic-data distribution. The resulting mel-spectrogram is converted into a time-domain waveform by a pretrained HiFi-GAN vocoder [23], which remains frozen during acoustic-model training.

### 3.3. Emotion–text decoupling attention module

**Fig 2:**
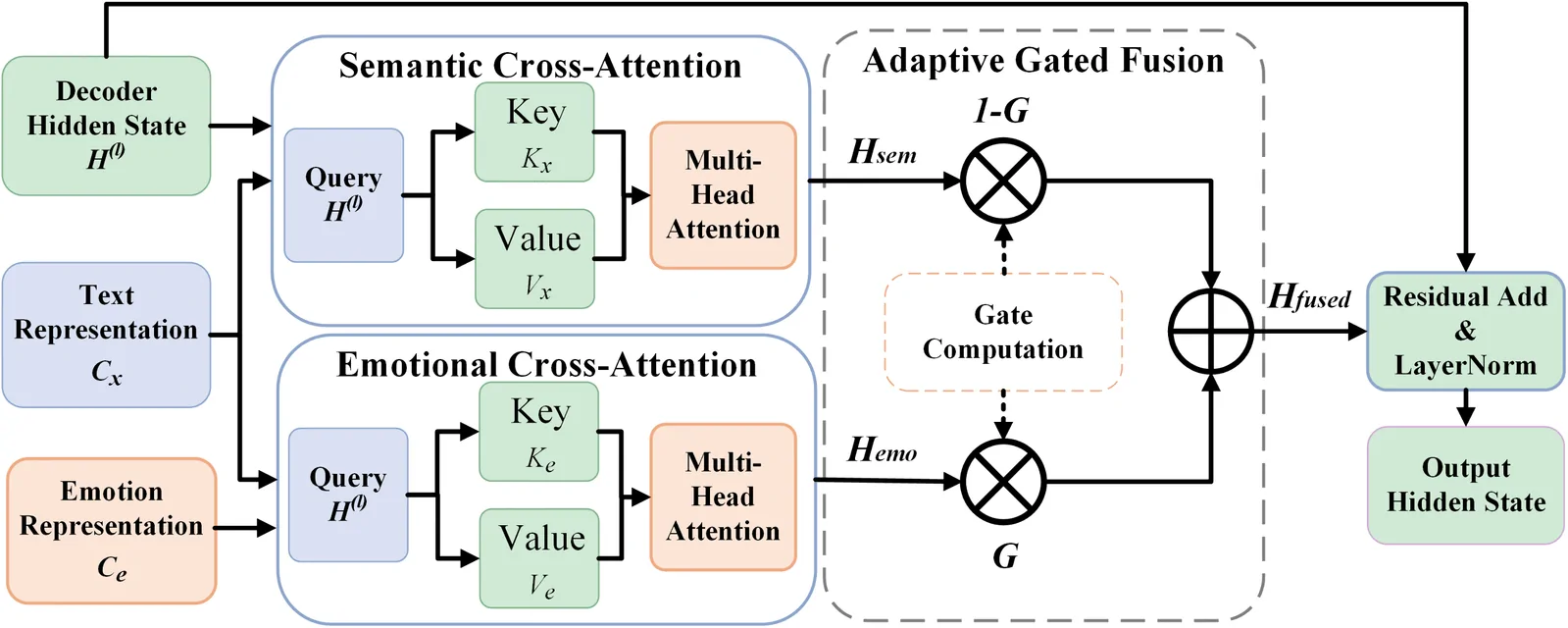
Internal structure of the ETDA module.

Textual and emotional conditions are related during emotional speech generation, but introducing them into the same attention operation may cause their representations to interact before condition-specific information has been sufficiently extracted. ETDA therefore separates the two attention computations and postpones their direct interaction until the fusion stage, as illustrated in Fig 2.

Let $H^{\left(l\right)}\in {\mathbb{R}}^{T\times D}$ denote the input hidden state of decoder layer *l*, where *T* is the acoustic-frame length and *D* is the hidden dimension. Let $c_{x}$ and $c_{e}$ denote the semantic and emotiona*l* representations. The decoder hidden state is linearly projected into two stream-specific query matrices, whereas the semantic and emotional condition representations are independently projected into their corresponding keys and values:


$$\begin{array}{cccccc} Q_{x} & =H^{\left(l\right)}W_{Q}^{x}, & K_{x} & =c_{x}W_{K}^{x}, & V_{x} & =c_{x}W_{V}^{x},\\ Q_{e} & =H^{\left(l\right)}W_{Q}^{e}, & K_{e} & =c_{e}W_{K}^{e}, & V_{e} & =c_{e}W_{V}^{e}. \end{array}$$ (4)

Here, $W_{Q}^{x}$, $W_{K}^{x}$, and $W_{V}^{x}$ denote the projection matrices of the semantic stream, whereas $W_{Q}^{e}$, $W_{K}^{e}$, and $W_{V}^{e}$ denote those of the emotional stream. The two sets of parameters are learned independently. The semantic and emotional representations retrieved by the two cross-attention streams are calculated as


$$\begin{array}{cc} H_{sem} & ={\mathrm{MHA}}_{x}(Q_{x},K_{x},V_{x}),\\ H_{emo} & ={\mathrm{MHA}}_{e}(Q_{e},K_{e},V_{e}),\\ \mathrm{Attention}(Q,K,V) & =\mathrm{softmax}\left(\frac{QK^{𝖳}}{\sqrt{d_{k}}}\right)V. \end{array}$$ (5)

Here, $d_{k}$ is the key dimension of each attention head. Each multi-head attention operation performs scaled dot-product attention across multiple heads, concatenates the head outputs, and maps the concatenated representation back to the decoder hidden dimension. Consequently, $H_{sem}$ and $H_{emo}$ have compatible dimensions for subsequent fusion.

The two branch outputs are combined through adaptive gated fusion:


$$\begin{array}{cc} G & =\sigma \left([H_{sem};H_{emo}]W_{g}+b_{g}\right),\\ H_{fused} & =(1-G)⊙H_{sem}+G⊙H_{emo}. \end{array}$$ (6)

In Eq. (6), $W_{g}$ and $b_{g}$ are learnable parameters, σ(·) is the sigmoid function, and ⊙ denotes element-wise multiplication. The gate *G* adjusts the relative contribution of the two streams according to the current decoder representation. A smaller gate value assigns relatively more weight to the semantic stream, whereas a larger value increases the contribution of the emotional stream.

The layer output is obtained through residual addition and layer normalization:


$$\begin{array}{c} H^{\left(l+1\right)}=\mathrm{LayerNorm}\left(H^{\left(l\right)}+H_{fused}\right). \end{array}$$ (7)

The residual pathway preserves the incoming decoder state while allowing the independently extracted semantic and emotional information to modify the current-layer representation.

### 3.4. Adaptive loss-threshold reweighting

**Fig 3:**
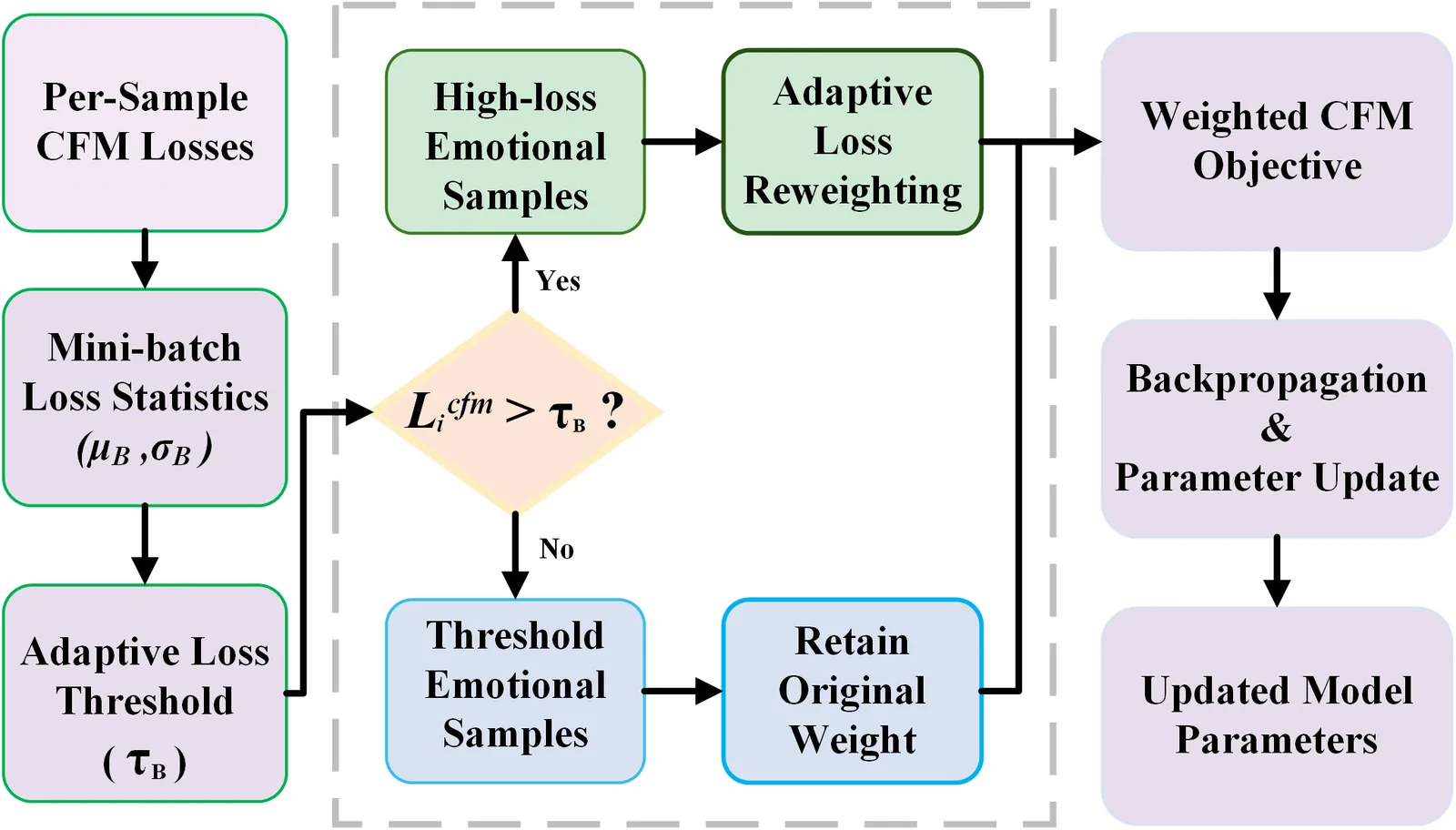
Workflow of Adaptive Loss-Threshold Reweighting during CFM training.

The standard CFM objective assigns the same coefficient to each sample when averaging the mini-batch losses. Although this formulation includes all samples, it does not provide additional optimization emphasis to high-loss emotional samples. Adaptive Loss-Threshold Reweighting therefore adjusts the sample contributions according to the loss distribution of the current mini-batch, as illustrated in Fig 3.

For a mini-batch containing *N* samples, the mean and standard deviation of the per-sample CFM losses are calculated as


$$\begin{array}{cc} \mu_{B} & =\frac{1}{N}\sum_{i=1}^{N}L_{i}^{CFM},\\ \sigma_{B} & =\sqrt{\frac{1}{N}\sum_{i=1}^{N}{\left(L_{i}^{CFM}-\mu_{B}\right)}^{2}}. \end{array}$$ (8)

An adaptive loss threshold is then determined by


$$\begin{array}{c} \tau_{B}=\mu_{B}+\alpha \sigma_{B}, \end{array}$$ (9)

where α controls the sensitivity of the threshold to the dispersion of the mini-batch losses. Because $\mu_{B}$ and $\sigma_{B}$ are recalculated for each mini-batch, the resulting threshold changes with the current training state rather than remaining globally fixed.

The weight of the *i*-th sample is defined as


$$\begin{array}{c} w_{i}=1+\gamma \max\left(0,L_{i}^{CFM}-\tau_{B}\right), \end{array}$$ (10)

where γ controls the magnitude of the additional weight. Samples satisfying $L_{i}^{CFM}\leq \tau_{B}$ retain the original weight $w_{i}=1$. Samples with losses above the threshold receive larger weights in proportion to the amount by which their losses exceed the threshold. No samples are discarded from the mini-batch objective.

The weighted CFM training objective is


$$\begin{array}{c} {ℒ}_{weighted}=\frac{1}{N}\sum_{i=1}^{N}w_{i}L_{i}^{CFM}. \end{array}$$ (11)

The mini-batch statistics, threshold, and weights are updated only during training and do not modify the inference procedure. The strategy identifies high-loss emotional samples solely based on their current per-sample CFM losses and does not rely on category-frequency or class-imbalance assumptions.

## 4. Experimental setup and results

### 4.1. Datasets

LJSpeech [24] is used for neutral speech training and in-domain evaluation, with 12,600 utterances used for training and 500 non-overlapping utterances reserved for testing. An emotional subset derived from ESD [25] is used for emotional training and zero-shot evaluation. The ESD-derived subset is partitioned at the speaker level, with 18 speakers used for training and two unseen speakers reserved for testing. The held-out speakers are excluded from emotion-classifier fine-tuning, clustering, and acoustic-model training, ensuring no speaker overlap between the training and test partitions.

To construct the fine-grained emotion space, a wav2vec 2.0 Base emotion classifier [22] removes utterances with posterior probabilities below 0.85. The remaining training utterances are clustered independently within each original coarse emotion category using K-Means [5] with Euclidean distance, based on utterance-level mean fundamental frequency and energy variance. Angry, Surprise, Sad, Happy, and Neutral are divided into 4, 3, 4, 2, and 3 clusters, respectively, yielding 16 fine-grained categories in total. Cluster centroids are estimated exclusively from the training partition, and each test utterance is assigned to the nearest training-derived centroid within its original coarse emotion category using the same acoustic features.

K-Means produces numerical cluster identifiers. Labels such as *Rage*, *Ecstasy*, and *Despair* are used as descriptive aliases for interpretation and do not constitute an independently validated or universal emotion taxonomy. The complete coarse-to-fine mapping, centroid statistics, category-level sample counts, and utterance-level assignments are available in the public GitHub and Figshare repositories associated with this study. Audio is converted into 80-dimensional mel-spectrograms, and phoneme alignments are obtained using the Montreal Forced Aligner [26]. The corpus statistics and the main configurations used to construct the fine-grained emotion space are summarized in Table 1.

**Table 1:** Corpus statistics and configurations for fine-grained emotion-space construction.

Corpus statistics						
Corpus	Usage	Language	Split	Speakers	Utterances	Duration (h)
LJSpeech	Neutral acoustic training	English	Training	1	12,600	23.08
LJSpeech	Neutral acoustic evaluation	English	Test	1	500	0.92
ESD-derived subset	Emotional speech training	English–Mandarin	Training	18	31,500	17.99
ESD-derived subset	Zero-shot evaluation	English–Mandarin	Test	2	3,500	2.03
**Fine-grained emotion-space construction**						
**Configuration**	**Value**	**Configuration**	**Value**			
Clustering algorithm	K-Means	Clustering distance metric	Euclidean			
K-Means n_init	10	Number of fine-grained classes	16			
K-Means max_iter	300	K-Means random seed	42			
K-Means initialization	k-means++	Confidence threshold	0.85			

### 4.2. Training setup

The experiments follow the corpus-specific data partitions described in Section 4.1. The proposed model and its ablation variants are trained on a single NVIDIA RTX 4090 GPU using AdamW [27]. The batch size is set to 32, and all models are trained for a fixed 120 epochs. The learning rate is linearly increased during the first 10 epochs and subsequently decayed from $2\times 10^{-4}$ to $1\times 10^{-6}$ using cosine annealing [28]. No validation set or early-stopping procedure is used. The checkpoint from the final training epoch is used for evaluation, and the test sets are used only for the final reported results. The remaining training configurations are summarized in Table 2.

**Table 2:** Hyperparameter configurations for the proposed speech synthesis framework.

Hyperparameter	Value	Hyperparameter	Value
Sampling rate	16 kHz	Filter channels	768
FFT size / window length	1024	Dropout probability	0.1
Hop length	256	Batch size	32
Mel-frequency bins (*F*)	80	Training epochs	120
ETDA attention heads	4	Learning-rate schedule	Cosine annealing
ETDA attention layers	6	Linear warmup phase	10 epochs
AdamW coefficients $\left(\beta_{1},\beta_{2}\right)$	0.8, 0.99	AdamW weight decay	$1\times 10^{-4}$
Initial/final learning rate	$2\times 10^{-4}$ / $1\times 10^{-6}$	Threshold sensitivity (α)	1.05
Reweighting scale (γ)	0.5	Training checkpoint	Final epoch

All baseline systems use the same corpus-specific training and test partitions, evaluation utterances, and acoustic preprocessing procedures. Model-specific architectural and optimization settings follow the corresponding official implementations unless otherwise stated. To maintain stylistic consistency, all reference prompts are standardized to 3.0 s by extracting the central segment of the source utterances. A common set of prompts is randomly sampled once from the held-out speaker partition and then kept identical across all systems to ensure fair evaluation.

### 4.3. Evaluation metrics and statistical analysis

Objective evaluation includes word error rate (WER), mel-cepstral distortion (MCD), and cosine speaker similarity (SIM), which measure pronunciation accuracy, spectral distortion, and speaker preservation, respectively. A pretrained Whisper model [29] transcribes each synthesized utterance. For Mandarin utterances, both the reference and Whisper-generated transcriptions are segmented into words using Jieba before WER computation, whereas English transcriptions are tokenized using whitespace. WER is calculated independently for each utterance and then averaged across the test set.


$$\begin{array}{c} WER=\frac{S+D+I}{N}\times 100\%, \end{array}$$ (12)

where *S* is the number of substituted words, *D* is the number of deleted words, *I* is the number of inserted words, and *N* is the total number of words in the reference text. Lower WER indicates higher pronunciation accuracy.

MCD measures the spectral distance between synthesized and ground-truth speech:


$$\begin{array}{c} MCD=\frac{10}{\ln10}\sqrt{\frac{2}{M}\sum_{m=1}^{M}{\left(C_{m}-{\hat{C}}_{m}\right)}^{2}}, \end{array}$$ (13)

where $C_{m}$ and ${\hat{C}}_{m}$ represent the *m*-th dimensional mel-frequency cepstral coefficients of the ground-truth and synthesized speech, respectively. Lower MCD indicates smaller spectral distortion. Cosine speaker similarity is calculated between embeddings extracted from the synthesized and reference utterances; higher SIM indicates better preservation of speaker characteristics.

Objective metrics are reported as means with two-sided 95% confidence intervals calculated using Student’s *t*-distribution based on the standard error of utterance-level scores across the test set. Pairwise differences between the proposed method and each baseline are assessed using two-sided Wilcoxon signed-rank tests [30] on paired utterance-level results. Holm correction [31] is applied to multiple comparisons, and an adjusted *p* < 0.05 is considered statistically significant.

Subjective evaluation is conducted using double-blind randomized listening tests. Separate 5-point Mean Opinion Score (MOS) scales are used to evaluate naturalness (NAT), speaker similarity (SIM), and emotional expressiveness (EMO) [32]. The MOS for each perceptual dimension is calculated as


$$\begin{array}{c} {MOS}_{d}=\frac{1}{N_{d}}\sum_{i=1}^{N_{d}}R_{i,d},\;d\in \{NAT,SIM,EMO\}, \end{array}$$ (14)

where $N_{d}$ is the number of valid ratings for dimension *d*, and $R_{i,d}$ is the *i*-th rating for that dimension.

Twenty bilingual listeners, including ten males and ten females aged 18–35 years, participated in the listening test. All listeners had pure-tone hearing thresholds of no more than 25 dB HL at 0.5, 1, 2, and 4 kHz, and none had professional experience in speech synthesis. Each listener evaluated exactly 48 stimuli, yielding 960 listener–stimulus evaluations in total. A balanced assignment was used to provide comparable numbers of ratings across systems and emotion categories, and a 5-s interval was inserted between consecutive stimuli. MOS results are reported as means with two-sided 95% confidence intervals calculated using Student’s *t*-distribution based on listener-level mean scores.

Pairwise differences are assessed using two-sided Wilcoxon signed-rank tests on listener-level paired scores [30], with the statistical significance threshold set at *p* < 0.05. Holm’s step-down correction is applied to adjust *p*-values for multiple pairwise comparisons [31].

### 4.4. Objective acoustic evaluation

Table 3 compares the proposed method with VITS [33], VALL-E [1], Matcha-TTS [19], NaturalSpeech 3 [10], CosyVoice [3], and StyleTTS 2 [21] under an utterance-disjoint in-domain setting on LJSpeech and a speaker-disjoint zero-shot setting on ESD. The Ground-Truth Mel + Vocoder condition is included as a reconstruction reference and is not treated as a trainable synthesis system.

**Table 3:** Objective evaluation results (mean ± 95% confidence intervals).

Dataset	Model	WER (%) ↓	MCD (dB) ↓	SIM ↑
LJSpeech	Ground-Truth Mel + Vocoder	1.15±0.03	2.12±0.02	0.982±0.005
	VITS	3.90±0.08	4.63±0.06	0.824±0.015
	VALL-E	5.62±0.12	5.22±0.08	0.810±0.018
	Matcha-TTS	3.46±0.06	4.35±0.05	0.852±0.011
	NaturalSpeech 3	3.18±0.05	4.24±0.05	0.873±0.012
	CosyVoice	3.05±0.04	4.15±0.04	0.885±0.010
	StyleTTS 2	2.95±0.04	4.10±0.04	0.892±0.010
	**Proposed**	2.82±0.04	3.95±0.03	0.917±0.008
ESD	Ground-Truth Mel + Vocoder	1.32±0.04	2.25±0.03	0.975±0.006
	VITS	6.48±0.14	5.32±0.09	0.765±0.022
	VALL-E	9.85±0.22	6.10±0.15	0.742±0.025
	Matcha-TTS	5.85±0.11	5.15±0.08	0.795±0.016
	NaturalSpeech 3	5.21±0.09	4.93±0.07	0.830±0.015
	CosyVoice	4.85±0.08	4.80±0.06	0.845±0.012
	StyleTTS 2	4.65±0.07	4.75±0.06	0.855±0.011
	**Proposed**	3.62±0.06	4.45±0.05	0.883±0.009

On LJSpeech, the proposed method obtains a WER of 2.82±0.04%, an MCD of 3.95±0.03 dB, and a SIM score of 0.917±0.008. The results are slightly more favorable than those of StyleTTS 2 and CosyVoice, which are the strongest external baselines on this corpus. This indicates that introducing fine-grained emotional conditions does not substantially reduce content preservation or speaker similarity under relatively stable acoustic conditions.

On ESD, all systems show weaker objective results than on LJSpeech, reflecting the increased difficulty associated with multi-speaker and emotional variation. The proposed method nevertheless maintains lower WER and MCD and higher SIM than the evaluated baselines, obtaining 3.62±0.06%, 4.45±0.05 dB, and 0.883±0.009, respectively. The differences are more visible than those observed on LJSpeech, particularly in comparison with Matcha-TTS, NaturalSpeech 3, and CosyVoice. Overall, the results suggest that the proposed method maintains linguistic content, spectral reconstruction, and speaker characteristics across the two evaluated corpora.

### 4.5. Subjective perceptual quality evaluation

Table 4 reports the subjective ratings for naturalness, speaker similarity, and emotional expressiveness. On LJSpeech, the proposed method obtains NAT and SIM scores of 4.65±0.05 and 4.60±0.06. These scores are slightly higher than those of StyleTTS 2 and CosyVoice, while remaining below the Ground-Truth Mel + Vocoder reference. The results are generally consistent with the objective evaluation and indicate favorable perceived naturalness and speaker preservation under standard acoustic conditions.

**Table 4:** Mean Opinion Score evaluation results (mean ± 95% confidence intervals).

Dataset	Model	NAT	SIM	EMO	*p*-value (EMO)
LJSpeech	Ground-Truth Mel + Vocoder	4.88±0.03	4.85±0.04	–	–
	VITS	4.34±0.06	4.28±0.07	–	–
	VALL-E	4.12±0.08	4.15±0.06	–	–
	Matcha-TTS	4.32±0.05	4.25±0.05	–	–
	NaturalSpeech 3	4.45±0.06	4.40±0.07	–	–
	CosyVoice	4.52±0.05	4.48±0.06	–	–
	StyleTTS 2	4.57±0.04	4.52±0.05	–	–
	**Proposed**	4.65±0.05	4.60±0.06	–	–
ESD	Ground-Truth Mel + Vocoder	4.82±0.04	4.78±0.05	4.86±0.04	–
	VITS	4.10±0.07	4.02±0.08	4.05±0.07	<0.001
	VALL-E	3.85±0.08	3.91±0.09	3.87±0.08	<0.001
	Matcha-TTS	4.15±0.06	4.08±0.07	4.15±0.06	<0.001
	NaturalSpeech 3	4.25±0.07	4.20±0.08	4.34±0.05	<0.001
	CosyVoice	4.37±0.06	4.35±0.06	4.45±0.05	0.034
	StyleTTS 2	4.42±0.05	4.37±0.07	4.37±0.06	0.018
	**Proposed**	4.58±0.06	4.52±0.05	4.64±0.04	–

On ESD, the proposed method achieves NAT, SIM, and EMO scores of 4.58±0.06, 4.52±0.05, and 4.64±0.04, respectively. StyleTTS 2 remains a strong external baseline for naturalness and speaker similarity, whereas CosyVoice obtains the strongest external emotional-expressiveness score. The proposed method maintains relatively favorable results across all three dimensions rather than improving only one perceptual property. These findings suggest that the proposed framework preserves naturalness and speaker characteristics while improving emotional expression to some extent.

### 4.6. Ablation studies

Table 5 evaluates the contributions of ETDA, adaptive gated fusion, the semantic and emotional attention streams, and Adaptive Loss-Threshold Reweighting. All variants retain the same data partitions and training settings. In the w/o Adaptive Gating variant, the learned gate is replaced by equal-weight element-wise fusion.

**Table 5:** Results of the ablation studies.

Dataset	Model variant	WER (%)	MCD (dB)	NAT	SIM	EMO	*p*-value (EMO)
LJSpeech	**Proposed**	2.82±0.04	3.95±0.03	4.65±0.05	4.60±0.06	–	–
	w/o Adaptive Gating	2.89±0.04	4.05±0.04	4.58±0.05	4.52±0.06	–	–
	w/o Reweighting	2.95±0.05	4.15±0.04	4.50±0.06	4.45±0.05	–	–
	w/o ETDA	3.23±0.06	4.31±0.05	4.42±0.05	4.38±0.06	–	–
	w/o Emotion Attention	2.90±0.04	4.13±0.04	4.48±0.05	4.46±0.05	–	–
	w/o Semantic Attention	3.42±0.07	4.22±0.05	4.45±0.06	4.40±0.06	–	–
ESD	**Proposed**	3.62±0.06	4.45±0.05	4.58±0.06	4.52±0.05	4.64±0.04	–
	w/o Adaptive Gating	3.74±0.06	4.58±0.05	4.46±0.06	4.41±0.06	4.52±0.05	0.046
	w/o Reweighting	3.85±0.07	4.98±0.06	4.40±0.07	4.35±0.05	4.42±0.06	0.012
	w/o ETDA	4.84±0.11	5.25±0.08	4.15±0.06	4.10±0.07	4.08±0.07	<0.001
	w/o Emotion Attention	3.91±0.08	4.80±0.06	4.22±0.05	4.18±0.06	4.35±0.05	<0.001
	w/o Semantic Attention	5.10±0.12	4.65±0.07	4.35±0.07	4.28±0.07	4.40±0.06	0.003

Replacing adaptive gated fusion causes relatively small but consistent changes on both datasets. On ESD, WER and MCD increase to 3.74±0.06% and 4.58±0.05 dB, while NAT, SIM, and EMO also decrease. This suggests that dynamically adjusting the two attention streams provides an additional benefit compared with fixed fusion, although the effect is smaller than that of removing ETDA entirely.

Removing ETDA produces broader changes across objective and subjective measures. The ESD results show higher WER and MCD together with lower NAT, SIM, and EMO. The two single-stream ablations exhibit different response patterns: removing Semantic Attention has a stronger effect on WER, whereas removing Emotion Attention produces a clearer reduction in emotional expressiveness. These observations are consistent, to some extent, with the intended roles of the two streams, while also showing that their effects are not limited to a single metric.

Removing Adaptive Loss-Threshold Reweighting mainly affects acoustic reconstruction and emotional expression on ESD. MCD increases from 4.45±0.05 dB to 4.98±0.06 dB, and EMO decreases from 4.64±0.04 to 4.42±0.06. The changes on LJSpeech are comparatively smaller, indicating that sample-level reweighting may be more relevant under emotionally variable conditions.

Taken together, the complete configuration provides the most balanced results. ETDA primarily changes how semantic and emotional conditions are processed, whereas Adaptive Loss-Threshold Reweighting changes how individual training samples contribute to optimization.

### 4.7. Emotional consistency under text-length expansion conditions

To evaluate emotional consistency as the amount of generated content increases, three paired text conditions are constructed from selected ESD test sentences. Ori. retains the original 8–12-word texts. Ex-I expands each text to 20–30 words by adding contextually consistent descriptions, while Ex-II further extends it to 35–45 words. The central topic, target emotion, and logical continuity are preserved during expansion, and direct repetition or changes in emotional polarity are excluded.

The reference utterance, speaker condition, emotion label, and synthesis settings remain unchanged across the three conditions. CosyVoice [3], the w/o ETDA variant, and the proposed method therefore use identical inputs and reference conditions. WER is used to evaluate content preservation. A frozen wav2vec 2.0-based emotion classifier [34] estimates target-emotion posterior probabilities from consecutive non-overlapping 3-s waveform segments.

Table 6 shows that WER increases as the texts are expanded for all three systems. The proposed method changes from 3.41±0.06% under Ori. to 3.92±0.09% under Ex-II, while the changes are more evident for CosyVoice and the w/o ETDA variant. This pattern suggests that increasing text length introduces additional difficulty in maintaining linguistic content.

**Table 6:** WER results under text-length expansion conditions.

Text condition	Metric	CosyVoice	w/o ETDA	Proposed
Original (8–12 words)	WER (%) ↓	4.46±0.07	4.17±0.09	3.41±0.06
Expanded-I (20–30 words)	WER (%) ↓	4.81±0.09	4.72±0.11	3.64±0.07
Expanded-II (35–45 words)	WER (%) ↓	5.29±0.12	5.64±0.14	3.92±0.09

**Fig 4:**
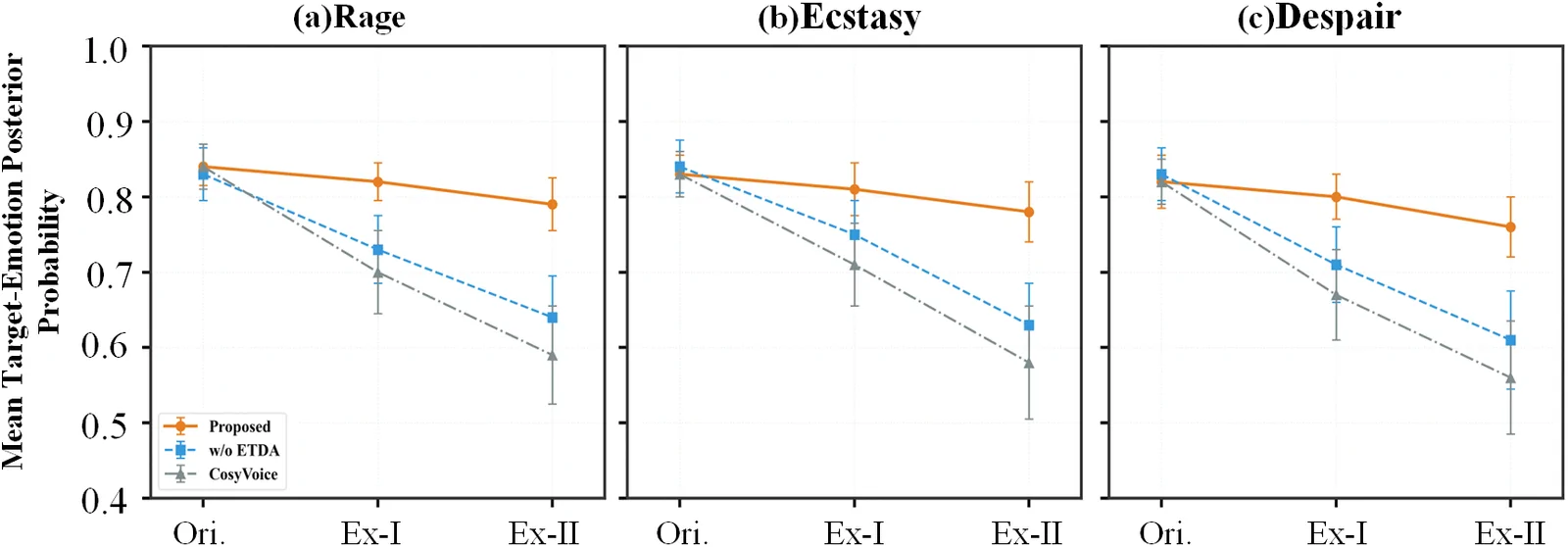
Mean target-emotion posterior probabilities under text-length expansion conditions. Mean target-emotion posterior probabilities are shown for the proposed method, the w/o ETDA variant, and CosyVoice under the original and two text-expansion conditions for Rage, Ecstasy, and Despair. Error bars denote the standard deviation across evaluation utterances.

Fig 4 shows a similar tendency in emotional expression. The three systems obtain relatively close posterior probabilities under Ori., but their trajectories separate under Ex-I and Ex-II. The proposed method retains comparatively higher values across Rage, Ecstasy, and Despair, particularly under Ex-II. Thus, the distinction between systems becomes clearer as more semantic content must be generated.

The joint WER and posterior-probability results indicate that text-length expansion affects both linguistic accuracy and emotional consistency. Under the evaluated conditions, the proposed method appears less sensitive to this increase, which is consistent, to some extent, with processing semantic and emotional conditions separately before adaptive fusion.

### 4.8. High-loss emotional sample analysis

A fixed high-loss emotional subset is used to further examine synthesis performance on samples with relatively large reconstruction losses. The subset is selected from the held-out ESD test set using the per-sample CFM losses produced by the w/o Reweighting variant. Within each of the Rage, Ecstasy, and Despair categories, the 20 samples with the highest losses are retained, producing a fixed subset of 60 utterances. The same texts, speakers, references, and emotion labels are used for all system comparisons.

Table 7 compares StyleTTS 2 [21], the w/o Reweighting variant, and the proposed method using MCD and MOS-EMO. The proposed method obtains lower MCD and higher MOS-EMO across all three emotion categories. The result is consistent across Rage, Ecstasy, and Despair rather than being concentrated in one category. The difference is somewhat more visible for Despair, where the external systems also obtain lower emotional-expressiveness ratings.

**Table 7:** Evaluation of acoustic reconstruction on high-loss emotional samples.

Emotion category	Metric	StyleTTS 2	w/o Reweighting	Proposed
Rage	MCD (dB) ↓	5.22±0.07	4.93±0.06	4.61±0.04
	MOS-EMO ↑	4.33±0.05	4.46±0.04	4.58±0.03
Ecstasy	MCD (dB) ↓	5.17±0.05	4.84±0.06	4.56±0.05
	MOS-EMO ↑	4.23±0.06	4.39±0.04	4.53±0.04
Despair	MCD (dB) ↓	5.33±0.06	4.96±0.07	4.62±0.06
	MOS-EMO ↑	4.16±0.07	4.34±0.06	4.52±0.04

The objective and subjective measures show the same overall direction. Lower MCD indicates that the generated spectra are closer to the references, whereas the higher MOS-EMO scores suggest that listeners perceive the target emotion more clearly. Their agreement provides broader support than either metric alone.

**Fig 5:**
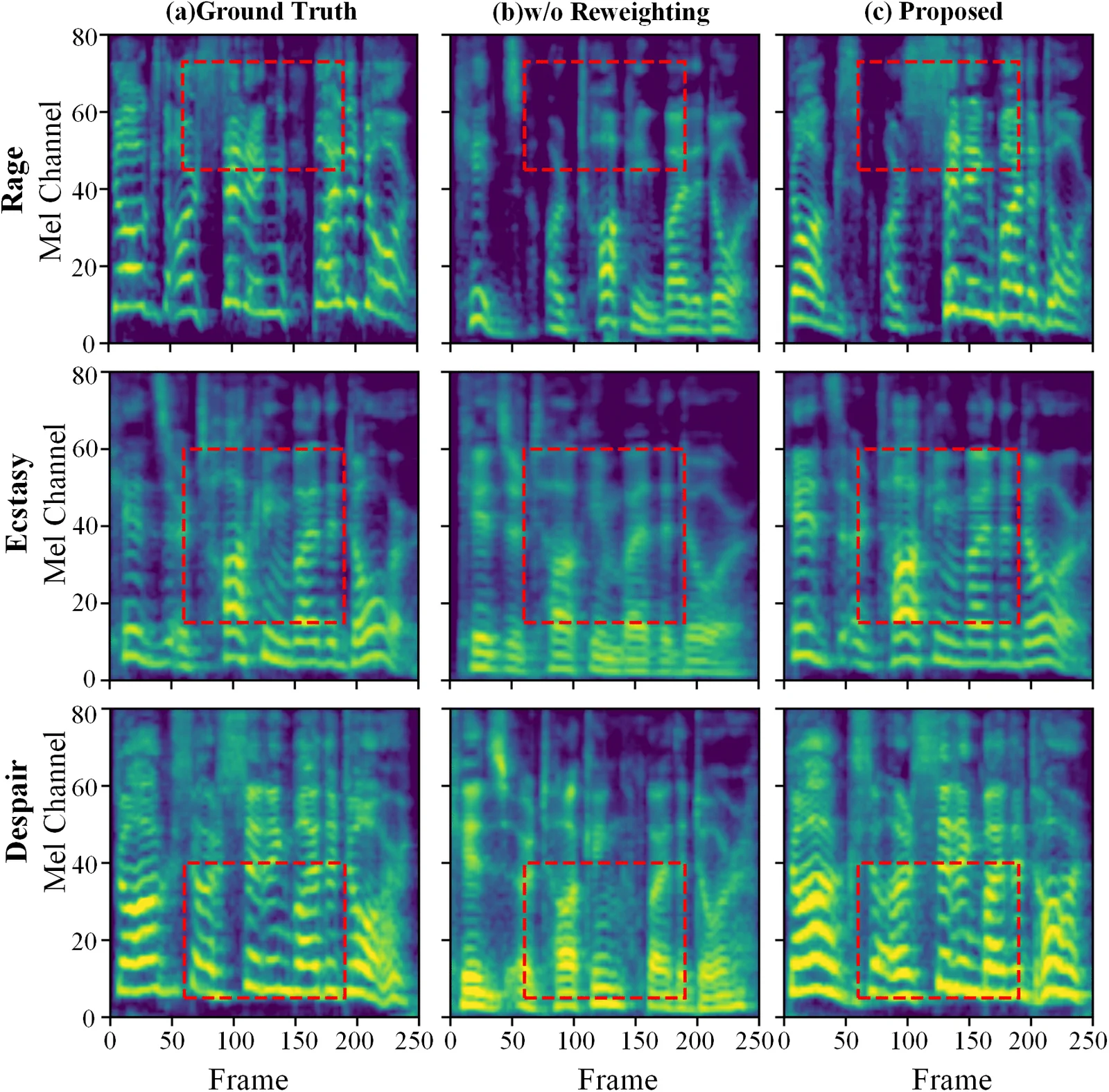
Mel-spectrogram comparison on representative high-loss emotional samples. Representative Ground-Truth, w/o Reweighting, and proposed-method mel-spectrograms are shown for Rage, Ecstasy, and Despair. The highlighted regions indicate local time–frequency structures used for qualitative comparison.

Fig 5 presents representative mel-spectrograms from the same subset. Compared with the w/o Reweighting variant, the proposed method retains more continuous local time–frequency patterns in several highlighted regions and appears closer to the Ground-Truth references. These findings suggest that Adaptive Loss-Threshold Reweighting may improve the reconstruction of high-loss emotional samples to some extent by increasing their contribution during training.

### 4.9. Visualization of fine-grained emotion representations

**Fig 6:**
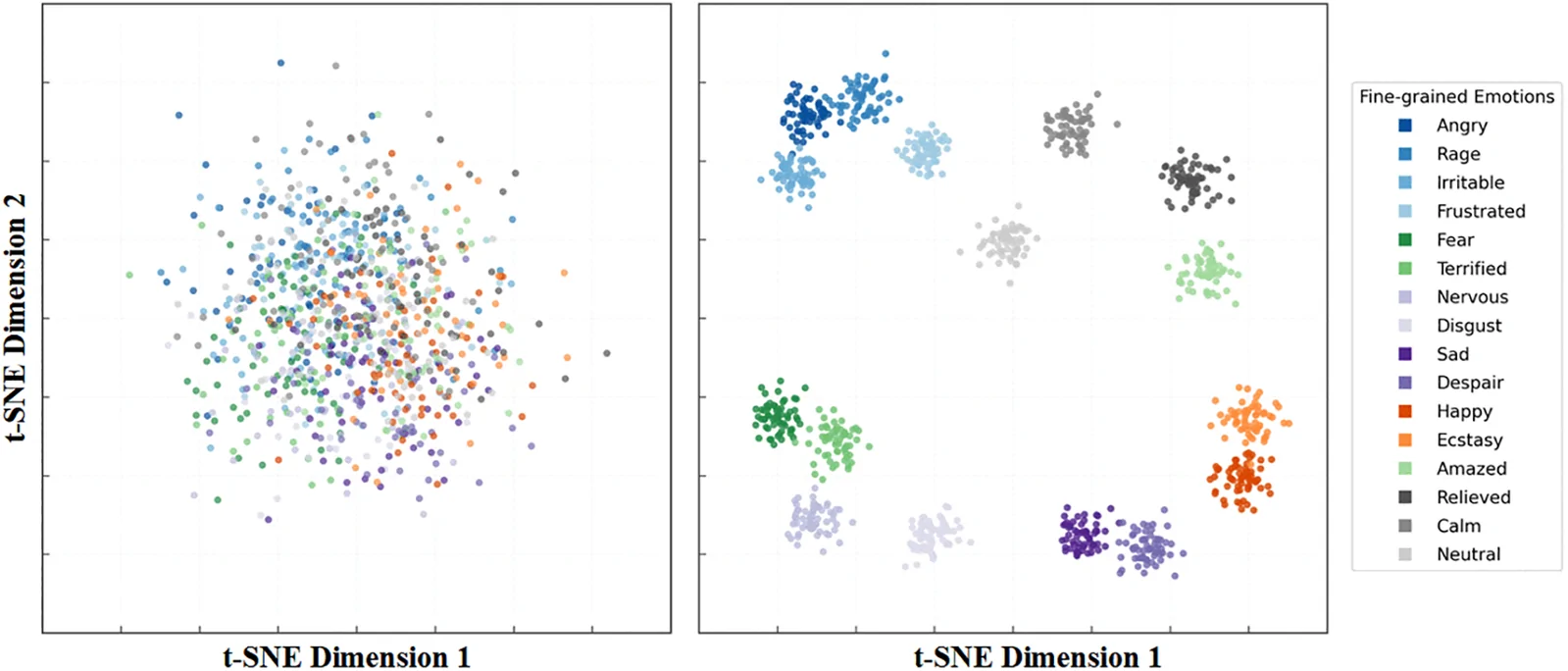
t-SNE visualization of the original acoustic features (left) and learned emotion embeddings (right).

To qualitatively examine the organization of the learned 16-class emotion space, t-SNE is applied to the utterance-level emotion representations extracted by the proposed method. Each point in Fig 6 represents one evaluation utterance, and different colors denote the 16 fine-grained emotion categories. The left panel presents the distribution of the original acoustic emotion features, where substantial overlap can be observed among different categories. The right panel presents the learned fine-grained emotion embeddings, in which several categories form relatively compact local clusters with clearer inter-category separation. Nevertheless, partial overlap remains among some acoustically related emotion categories. Therefore, the visualization is interpreted only as a qualitative illustration of representation-space organization rather than formal evidence of complete separability or perceptual independence.

## 5. Discussion

The objective and subjective results show that the proposed method generally obtains more favorable results than the evaluated synthesis baselines on LJSpeech and ESD, with larger differences under the fine-grained emotional conditions of ESD. These findings suggest that synthesis quality depends not only on the generative formulation, but also on how semantic and emotional conditions are processed and how high-loss emotional samples contribute to training. Nevertheless, the remaining gap from the Ground-Truth Mel + Vocoder reference indicates that the acoustic-generation stage still introduces additional errors.

The ablation and text-length-dependent results support the contribution of ETDA. Removing ETDA or replacing adaptive gated fusion with a fixed fusion operation degrades both objective and perceptual performance. Moreover, the w/o ETDA variant and CosyVoice show clearer decreases in target-emotion posterior probability under the expanded-text conditions, whereas the proposed method retains comparatively higher values. These results suggest that parallel semantic and emotional attention followed by adaptive fusion is beneficial for linguistic-content preservation and emotional consistency as the amount of generated content increases. However, classifier posterior trajectories should be interpreted as external indicators of emotional consistency rather than direct measurements of internal condition interference.

Adaptive Loss-Threshold Reweighting also improves the treatment of high-loss emotional samples. Unlike uniform averaging, the strategy increases the contribution of samples whose individual CFM losses exceed the mini-batch threshold. Its removal leads to higher spectral distortion and lower perceptual emotion scores, while the fixed high-loss subset and mel-spectrogram comparisons provide additional supporting evidence. These results suggest that, under the evaluated conditions, sample-level reweighting can improve the reconstruction of high-loss emotional samples to some extent.

The present study has several limitations. CFM inference still requires multiple numerical integration steps, which may limit real-time deployment. The 16-class emotion space is derived from a specific corpus and may depend on the classifier, acoustic features, and clustering settings. In addition, reference-prompt duration and local tone–emotion interaction, particularly in Mandarin, require more systematic evaluation. Future work will examine reduced-step generation, broader cross-corpus validation, naturally occurring long-form emotional speech, and finer prosodic control.

## 6. Conclusion

This study proposes a Conditional Flow Matching framework for fine-grained zero-shot emotional speech synthesis by integrating Emotion–Text Decoupling Attention (ETDA) and Adaptive Loss-Threshold Reweighting. The proposed framework jointly improves condition modeling and training optimization while introducing a 16-class fine-grained emotion space derived from classifier filtering and acoustic clustering.

Experiments on LJSpeech and ESD demonstrate generally consistent improvements over representative zero-shot speech synthesis baselines in both objective and subjective evaluations. The ablation studies indicate that ETDA, adaptive gated fusion, and Adaptive Loss-Threshold Reweighting each make positive contributions to the overall performance. Furthermore, analyses based on text-length expansion, high-loss emotional samples, and t-SNE visualization provide additional evidence supporting the proposed framework under the evaluated conditions.

Overall, the proposed framework improves linguistic-content preservation, emotional expression, and acoustic reconstruction for fine-grained zero-shot emotional speech synthesis under the evaluated conditions. Future work will focus on more efficient CFM inference, broader cross-corpus validation, naturally occurring long-form emotional speech, and finer prosodic control.
